# Fabrication and Characterization of Carbon Fiber Reinforced Plastics Containing Magnetostrictive Fe-Co Fibers with Damage Self-Detection Capability

**DOI:** 10.3390/s19224984

**Published:** 2019-11-15

**Authors:** Kenichi Katabira, Hiroki Kurita, Yu Yoshida, Fumio Narita

**Affiliations:** Department of Materials Processing, Graduate School of Engineering, Tohoku University, Aoba-yama 6-6-02, Sendai 980-8579, Japan; kenichi.katabira.s2@dc.tohoku.ac.jp (K.K.); kurita@material.tohoku.ac.jp (H.K.); yu.yoshida.p8@dc.tohoku.ac.jp (Y.Y.)

**Keywords:** mechanical design, CFRP, Fe-Co fiber, magnetostrictive response, sensors, structural health monitoring

## Abstract

Carbon fiber reinforced plastic (CFRP) is an excellent choice in the areas where weight reduction is important and multi-functionalization of CFRP, especially by adding sensor capabilities, is a promising approach to realize lightweight battery-free devices in structural health monitoring (SHM). In this study, we fabricated hybrid CFRP with Fe-Co fibers and evaluated the inverse magnetostrictive response characteristics. It was shown that the measured magnetic flux density of the CFRP fluctuates in response to cyclic bending load. It was also revealed that our Fe-Co fiber inserted CFRP has damage self-sensing ability. In addition, it seems that the optimization of design and more experimental and numerical investigation improves the capability of the hybrid CFRP with Fe-Co fiber as sensor composite materials.

## 1. Introduction

Structural health monitoring (SHM) is an emerging research area with applications in critical structures, for example, aerospace, civil, and mechanical structures [1]. The sensor types for SHM include strain gage, fiber Bragg gratings, piezoelectric materials and so forth [2]. The monitoring systems using them have been developed to evaluate various damage modes in a structure with minimal cost [3].

The use of inverse magnetostriction is a good approach for SHM, and low-cost materials with large magnetostriction are necessary [4]. Tb*_x_*Dy_1−*x*_Fe*_y_* (0.27 ≤ *x* ≤ 0.30, 1.9 ≤ *y* ≤ 2.0), known as Terfenol-D, has giant magnetostriction and low magneto-crystalline anisotropy [5]. Calkins et al. have reviewed that Terfenol-D can be widely utilized as a sensor [6]. Fe-Ga alloy, known as Galfenol, also exhibits large magnetostriction, and Hein et al. have studied its characteristics as a toque sensor [7]. Recently, Li et al. have studied (Fe_83_Ga_17_)_99.4_B_0.6_ wires of 0.5 mm diameter which were prepared by hot forging, rolling and combining hot and cold drawing [8]. They have shown the possibility to improve the working temperature of the magnetostrictive displacement sensors. Several researchers have investigated the magnetostrictive polymer composites with Terfenol-D and Galfenol as sensors [9,10,11].

Fe-Co alloys are suitable for sensor applications due to rich elements and lower cost compared with Terfenol-D and Galfenol and have many favorable characteristics that allow easy fabrication, high strength, ductility, and excellent workability [4]. Yamazaki et al. have characterized Fe-Co wire and designed a magnetostrictive sensor for SHM [12,13]. These characteristics enable development of magnetostrictive composites [14]. Recently, Katabira et al. have fabricated the magnetostrictive fiber dispersed polymer matrix composites, in which Fe-Co fibers were woven into a polyester fabric, and examined their sensor performance [15].

The applications of carbon fiber reinforced plastics (CFRPs) have been investigated and applied in industry, sports goods, aerospace, and other fields, because of the excellent mechanical properties [16,17]. Recently, piezoelectric hybrid CFRP laminates have been developed by embedding potassium sodium niobite (KNN) nanoparticle filled epoxy interlayer, and the composite samples have been successfully polarized [18]. It has been shown that the output voltage of the lead-free KNN nanoparticle filled CFRP is approximately 70 mV and is larger than that of the barium titanate nanoparticle filled CFRP. In the Terfenol-D/CFRP composites, however, literature studies of magnetostrictive properties are sparse and inconclusive [19,20].

In this study, we aim to develop magnetostrictive hybrid CFRP for sensors. CFRP laminate was fabricated by embedding magnetostrictive Fe-Co fibers, and the composite sample was successfully cured. The bending test was then performed to measure the magnetic flux density change of the CFRP laminate. The damage self-sensing of the CFRP laminate was investigated and discussed in detail.

## 2. Experimental Procedure

**Figure 1:**
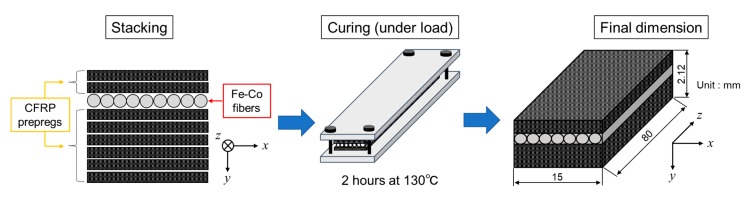
Schematic for specimen preparation.

We employed the Fe-Co continuous fibers with a diameter of 0.2 mm, and the composition is Fe_29_Co_71_. The Fe-Co fibers were inserted between CFRP prepregs (F6343B-05P, Toray Industries, Inc., Tokyo, Japan), and Young’s modulus of Fe-Co fiber and carbon fiber in the prepreg are approximately 182 and 230 GPa, respectively. They were cut to obtain the final composite with Fe-Co fibers in the longitudinal (*z*-)direction of the specimen as shown in Figure 1. Warp direction is parallel to the longitudinal direction of the specimen, and fill direction is parallel to transverse (*x*-)direction. After cutting, CFRP prepregs (CF) and Fe-Co fibers (Fe-Co) were stacked so that the layers became [CF]_2_/Fe-Co/[CF]_6_. A subscript is the number of CFRP prepregs. The specimen was then cured for 2 h at 130 °C. The final dimension is also shown in Figure 1. The Fe-Co fibers in the sample were located above the midplane. Katabira et al. have reported that the design improves sensor performance under the bending test [15].

**Figure 2:**
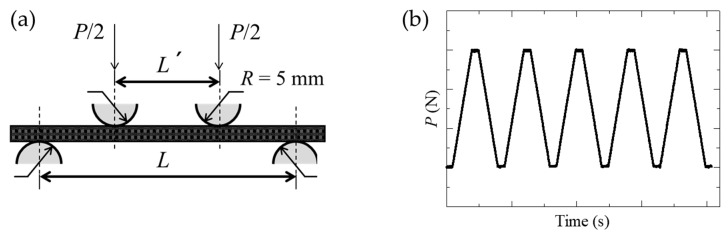
Experimental setup; (**a**) four-point bending test, and (**b**) a cyclical test program.

**Figure 3:**
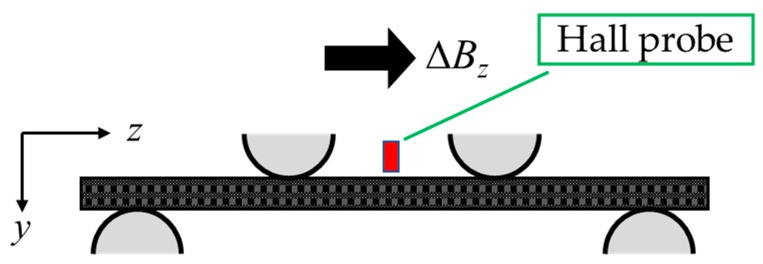
Location of hall probe and measurement direction.

Four-point bending tests were carried out on the CFRP with Fe-Co fibers by using Autograph (AG-50kNXD, Shimadzu Corporation, Kyoto, Japan). Figure 2a shows the experimental setup of four-point bending tests. The load and support span length were *L*’ = 22 and *L* = 66 mm, respectively. A cyclical test program was used as shown in Figure 2b, and the stress rate was 10 MPa/s. Gaussmeter (GM-4002, Denshijiki Industry Co., Ltd., Tokyo, Japan) was used for the measurement of magnetic flux density change Δ*B*, which is increment or decrement of the magnetic flux density under the load *P*. The measurement range was set to ±4 mT, which was the minimum range of the Gaussmeter and decided by a preliminary experiment. The location of hall probe was the center of the specimen on the upper side, and Δ*B_z_* was measured, which was magnetic flux density change in the *z*-direction (see Figure 3).

## 3. Results and Discussion

**Figure 4:**
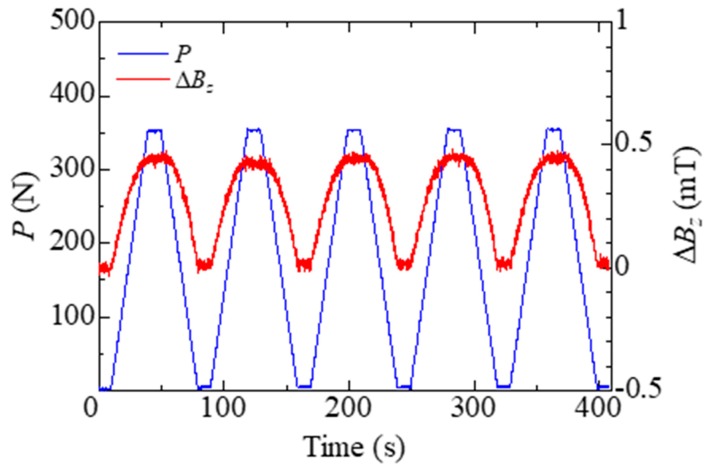
Load *P* and magnetic flux density change Δ*B_z_* versus time.

Figure 4 shows the load *P* and magnetic flux density change Δ*B_z_* versus time. The magnetic flux density fluctuated in response to the cyclic load. In addition, the magnetic flux density changes due to the maximum load in each cycle being much the same. This result implies that the magnetostrictive hybrid CFRP with Fe-Co fibers can detect bending stress. A similar effect may also occur if we apply a compressive load on fibers in *x*- or *y*-direction. However, Fe-Co fiber has a columnar structure due to continuous drawing, and the domains align along the longitudinal direction. We then assumed that the compressive load on fibers in the *z*-direction is more effective than that in other directions.

**Figure 5:**
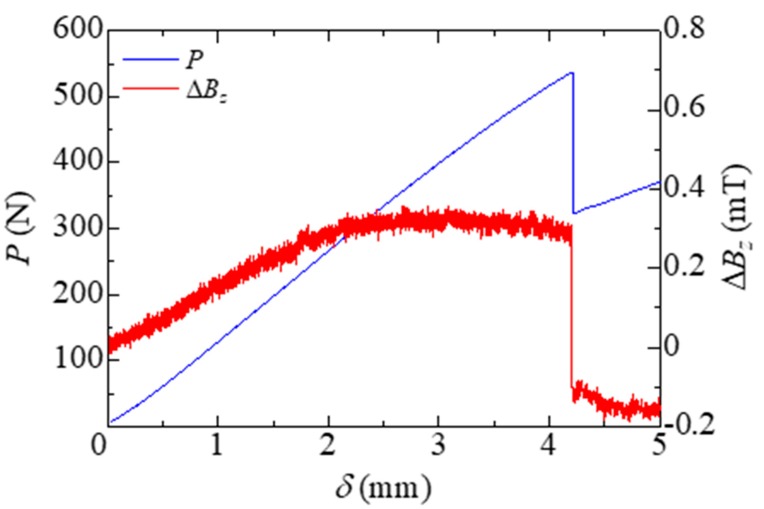
Load *P* and magnetic flux density change Δ*B_z_* versus load point displacement *δ*.

**Figure 6:**
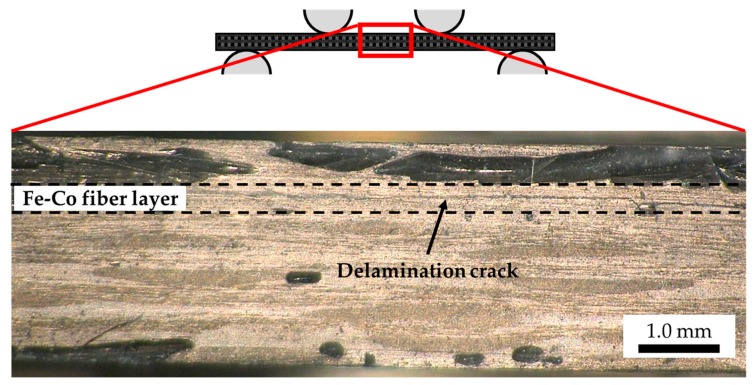
Edge view of the specimen.

After the cyclic test, we also investigated the damage self-sensing of the specimen. Figure 5 shows the load *P* and the magnetic flux density change Δ*B_z_* as a function of load point displacement *δ*. The load decreased just beyond the peak load drastically, and the specimen failed catastrophically. Figure 6 shows the edge view of the specimen. Since the delamination crack around the Fe-Co fiber layer was observed, the sharp drop of the load is taken as an indication that delamination damage has occurred. The magnetic flux density change-displacement behavior displayed a drastic decrease equally in delamination damage. Therefore, the delamination damage initiation allows being monitored through magnetic flux density measurements. On the other hand, in order to plan applications in the field of SHM, it is necessary to quantify the relationship between the damage situation and the amount of magnetic flux density change. This is a challenging research area, and sooner or later some progress will be made.

## 4. Conclusions

We fabricated hybrid CFRP with magnetostrictive Fe-Co fibers and evaluated the magnetic flux density change of the CFRP by a four-point bending test. The magnetic flux density fluctuated due to cyclic bending load and delamination damage. The results implied that measuring magnetic flux density probably allows the identifying of applied load and damage. Therefore, we believe that this study contributes to developing expectancy of lightweight, robust, high-performance stress sensors. For example, the hybrid CFRP with Fe-Co fibers might be used as a stress sensor for SHM in aerospace systems because the reliability is crucial to operating them safely. Certainly, the integration of Fe-Co fibers will significantly degrade the hybrid CFRP, which is a major issue in the future. We will consider effective solutions such as the use of finer Fe-Co fibers and the choice of the optimal matrix resin. Work in this area is currently being pursued. Consequently, it seems that the optimization of the design of the hybrid CFRP with Fe-Co fiber and more experimental and numerical investigation allows the improvement of sensor efficiency.
